# One-Step RT-qPCR for Viral RNA Detection Using Digital Analysis

**DOI:** 10.3389/fbioe.2022.837838

**Published:** 2022-03-07

**Authors:** Hyuna Park, Wonjong Jung, Hyeongseok Jang, Kak Namkoong, Kwon-Young Choi

**Affiliations:** ^1^ Department of Environmental Engineering, College of Engineering, Ajou University, Suwon-si, South Korea; ^2^ Device Research Center, Advanced Sensor Lab, Samsung Advanced Institute of Technology, Samsung Electronics Co.Ltd., Suwon-si, South Korea

**Keywords:** thermolysis, RT-qPCR, virus detection, one-chip digital PCR, multiplex detection

## Abstract

The rapid detection of viruses is becoming increasingly important to prevent widespread infections. However, virus detection *via* reverse transcription-quantitative polymerase chain reaction (RT-qPCR) is time-consuming, as it involves independent nucleic acid extraction and complementary DNA synthesis. This process limits the potential for rapid diagnosis and mass analysis, which are necessary to curtail viral spread. In this study, a simple and rapid thermolysis method was developed to circumvent the need for extraction and purification of viral RNA. The developed protocol was applied to one-chip digital PCR (OCdPCR), which allowed thermolysis, RT, and digital PCR in a single unit comprising 20,000 chambers of sub-nanoliter volume. Two viruses such as tobacco mosaic virus and cucumber mosaic virus were tested as model viral particles. First, the temperature, exposure time, and template concentration were optimized against tobacco mosaic viral particles, and the most efficient conditions were identified as 85°C, 5 min, and 0.01 μg/nL with a cycle threshold of approximately 33. Finally, the OCdPCR analysis yielded 1,130.2 copies/µL using 10^−2^ μg/nL of viral particles in a 30 min thermolysis-RT reaction at 70°C. This novel protocol shows promise as a quick, accurate, and precise method for large-scale viral analysis in the future.

## 1 Introduction

The global spread of the coronavirus disease since late 2019 has resulted in the infection of over 190 million people worldwide, with more than 4.09 million fatalities, as of 22 July 2021 (World Health Organization, https://www.who.int). At several points during the pandemic, the number of infected individuals has rapidly increased due to small-scale infections and mutated viruses. Although recent trends have shown signs that the severity of the current pandemic is declining, there are growing concerns regarding similar situations in the future.

Along with the development of vaccines and treatments against virus, the diagnosis of viral infections using high-speed and accurate ribonucleic acid (RNA) detection assays is paramount, as various studies have reported (Brown et al., 2021; Garg and Garg, 2021; Howe et al., 2021; Ibrahimi et al., 2021; Luethy and Johnson, 2021; Van Rijn and Boonstra, 2021; Yuan et al., 2021). For example, an integrated microfluidic system with reverse transcription-polymerase chain reaction (RT-PCR) has been developed for the rapid detection of influenza A viruses (Shen et al., 2019). In addition, several assays and sensors for detection of pathogens such as virus have been developed, including quantitative RT-PCR (RT-qPCR) using loop-mediated isothermal amplification, magnetic bead-based RNA extraction for rapid large-scale testing, and electrode-based electrochemical immunosensing (Klein et al., 2020; Nagura-Ikeda et al., 2020; Fabiani et al., 2021; Taki et al., 2021). A fundamental limitation of the current diagnostic analysis methods of viral pathogens, however, is the reliance on two consecutive enzyme reactions: reverse transcription (RT), followed by polymerase chain reaction (PCR). This requires labor-intensive laboratory-based protocols to isolate viral RNAs and amplify deoxyribonucleic acid (DNA). At the laboratory level, it is difficult to achieve desirable accuracy and precision in viral RNA detection and diagnosis due to time and resource limitations. However, in a pandemic situation, allocation of necessary resources is prioritized, allowing for large-scale development of diagnostic methods.

Although RT-PCR is an accurate method for detecting viruses, certain disadvantages do exist (Van Rijn and Boonstra, 2021). The essential steps of isolating RNA from a viral sample and synthesizing complementary DNA (cDNA) through an RT reaction, then amplifying this cDNA using PCR, are routinely conducted manually (Garg and Garg, 2021; Voon et al., 2021; Yuan et al., 2021). In general, a total reaction time of at least 4 h is required to isolate viral RNA from biological samples, synthesize cDNA, and perform PCR according to the manufacturer protocols (Arevalo-Rodriguez et al., 2020; Ibrahimi et al., 2021). Moreover, conventional RT-qPCR analyses rely on 96- or 384-well plates, limiting high-throughput sample analysis in the case of mass infection. This can cause a shortage of commercial kits make and virus detection and diagnosis difficult. Additionally, the RT and PCR reactions differ in terms of optimum reaction conditions and constituents, such as solution pH, temperature, and concentration, and the buffer used. Although nucleic acid extraction and isolation kits are commercially available, they are expensive and time-consuming. For example, commercial viral RNA isolation kits require a lysis step involving chemical treatment, followed by a concentration step using an elution buffer (Li et al., 2015). This protocol limits large-scale sample analysis and rapid diagnosis (Tang et al., 2013; Abdallah et al., 2020; Ulloa et al., 2020). Additionally, the lysis buffers provided in commercial kits contain reagents such as RNase inhibitors for membrane lysis and RNA stabilization (Tosh et al., 1997; Li et al., 2015; Zucha et al., 2020). As RNA is relatively unstable during heating and hydrolysis, certain conditions can result in sample degradation by RNase, reducing the concentration of the RNA template and producing low amplification results (Campos et al., 2021; Rattanachaisit et al., 2021; Schoenmaker et al., 2021).

Due to the rapid spread of viral pathogens, precise, large-scale detection with few false positives and a high true negative rate is an important factor in diagnostic performance. Moreover, determining the precise concentration of viral RNA is the most critical factor in diagnosis, and depends on the absolute number of RNA copies in the initial analytical volume. This is challenging and limits the application of conventional RT-qPCR analysis. Therefore, a sensitive PCR technique that can analyze viral RNA with precise and repeatable outputs is required (De Boer et al., 2011). Recent digital PCR (dPCR) technology enables the precise analysis of nucleic acids, and are capable of absolute quantification of viral load regardless of the availability of reference RNA (Basu, 2017; Cristiano et al., 2021; Karon et al., 2021; Martin et al., 2021; Rattanachaisit et al., 2021; Yi Han Tan et al., 2021). dPCR can provide not only unparalleled precision by splitting the sample into tens of thousands of partitions and analyzing these each using microfluidic technology, but also low-level viral RNA detection even in the presence of inhibitors, by offering nanoscale reaction environments suitable for one-step thermolysis RT-qPCR. dPCR works by splitting a DNA or cDNA sample into several separate, parallel PCR reactions. After PCR analysis, the negative fraction can be used to generate an absolute number of target molecules in a sample, without standards or endogenous controls. Of course, the price of digital PCR equipment and consumable chips should be considered, but basically, RNA isolation and purification steps can be omitted, enabling analysis in a shorter time when RT and PCR reactions are performed in one pot. There is no need to rely on reference materials or standards, and it has the advantages of high resistance to inhibitors and excellent analysis ability for complex mixtures (Basu, 2017; Yi Han Tan et al., 2021).

A potential means to overcome common diagnostic limitations is to directly use a viral particle as an RT reaction template, bypassing the RNA isolation step. Additional treatment is necessary because the isolation of viral RNA templates for cDNA synthesis requires disruption of the viral membrane. This study presents a protocol to obtain viral RNA using simple thermolysis, in which viral RNA was directly utilized as an RT reaction template. Tobacco mosaic virus (TMV) and cucumber mosaic virus (CMV) were used as model virus particles (Knight, 1955; Du et al., 2013). Furthermore, a one-step direct RT-qPCR method was developed to detect viruses within a single PCR tube without the need for separate chemical treatment or purification. This protocol was applied to a one-chip digital PCR, which showed high accuracy and precision. As biological risks such as COVID-19 and other pathogens continue to be reported, it is necessary to prepare rapid identification protocols. This study provides an important reference for the development of rapid, precise, and high-throughput analysis methods for viral detection.

## 2 Materials and Methods

### 2.1 Virus Information

The tobacco mosaic virus (*Tobamovirus,* Accession No. PV-000806) was isolated from *Petunia hybrida* (Niehl et al., 2014), and cucumber mosaic virus (*Cucumovirus,* Accession No. PV-000302) was isolated from *Nicotina tabacum* (NCBI:txid4097) (Alonso-Prados et al., 1998). Viral particles were provided by the Plant Virus GenBank, Seoul, South Korea.

### 2.2 Preparation of Leaf Samples for Pre-Treatment

Viral particles were added to a sterilized 0.01 M potassium phosphate buffer (pH 7.0) and mixed thoroughly immediately prior to use without centrifugation in order to include debris in the assay sample. Powdered viral RNA samples were used as templates for chemical purification or thermal treatment. The concentration of infected leaves was expressed in µg/nL, using the weight of dried leaves in µg/volume of phosphate buffer in nL.

### 2.3 Pre-Treatment of Viral Particles for RT and RT-PCR

The Viral Gene-spin™ Viral DNA/RNA Extraction Kit (Promega, Madison, Wisconsin, United States) was supplied by Intron Biotechnology, South Korea. RNA was extracted according to manufacturer instructions. For thermolysis, ground leaves were placed in a heat block with varying temperature from 50 to 95°C for an appropriate time between 5 and 10 min.

### 2.4 Synthesis of cDNA

cDNA was synthesized from purified viral RNA or RNA in a heat-degraded leaf sample using two different commercial kits at room temperature. At 50°C, The TOPscript cDNA synthesis kit (Enzynomics Daejeon, South Korea) was used, while the RocketScript™ Reverse transcriptase (Bioneer, Daejeon, South Korea) was used at 70°C. Synthesis procedures were carried out according to manufacturer protocols.

### 2.5 PCR Conditions and Detection of TMV and CMV

Target genes for each of the two viruses were amplified using TOPReal™ qCPR 2x premix (Enzynomics) and the StepOnePlus Real-Time PCR System (Thermo Fisher Scientific, Waltham, MA, United States). Specific primer pairs were used to amplify TMV and CMV: 5′-CGA​CAT​CAG​CCG​ATG​CAG​C-3′ and 5′-ACC​GTT​TTC​GAA​CCG​AGA​CT-3′ were used for the forward, and 5′-ACC​GTT​TTC​GAA​CCG​AGA​CT-3′ and 5′-TAC​TGA​TAA​ACC​AGT​ACC​GGT​GA-3′ for the backward, respectively. Reverse transcriptase was mixed with the PCR premix and used in RT-PCR, and the temperature for RT was added to the front end of the PCR thermal cycle. For the one-step direct method, ground leaves were placed into the RT-PCR solution and the reaction was carried out for a total of 40 cycles. The PCR protocol was as follows: 10 s at 95°C for denaturation, 15 s at 60°C for annealing, and 30 s at 72°C for extension. The temperature for the combined thermolysis and RT reaction was 50°C.

### 2.6 Preparation for One-Chip Digital RT-PCR

CMV particles were added to a premix containing reverse transcriptase, primers, and a probe with master mix containing DNA polymerase, dNTP, buffer (Tris-HCl, KCl, MgCl_2_, pH8.5), etc. The reverse transcriptase and master mix used were RocketScript™ reverse transcriptase, RNase H minus (Bioneer), and QuantStudio™ 3D Digital PCR Master Mix v2 (Thermo Fisher Scientific), respectively. The prepared reaction solution containing CMV particles was injected into a loading blade, loaded onto a digital PCR chip (QuantStudio™ 3D Digital PCR 20 K Chip Kit v2, Thermo Fisher Scientific) comprising sub-nanoliter-sized chambers using a QuantStudio™ 3D Digital PCR Chip Loader (Thermo Fisher Scientific) and sealed.

### 2.7 Digital RT-PCR Conditions and Detection of CMV

For digital RT-PCR, The temperature for thermolysis and RT were conducted at 70°C. PCR steps included 40 cycles of 15 s at 96°C, 30 s at 56°C, and 30 s at 72°C. After the reaction, the fluorescence intensity of each sub-nL-sized chamber in a chip was measured using a QuantStudio™ 3D Digital PCR instrument (Thermo Fisher Scientific) and statistically analyzed based on Poisson distribution.

## 3 Results and Discussion

### 3.1 Evaluation of TMV Detection Through RT and PCR

**FIGURE 1:**
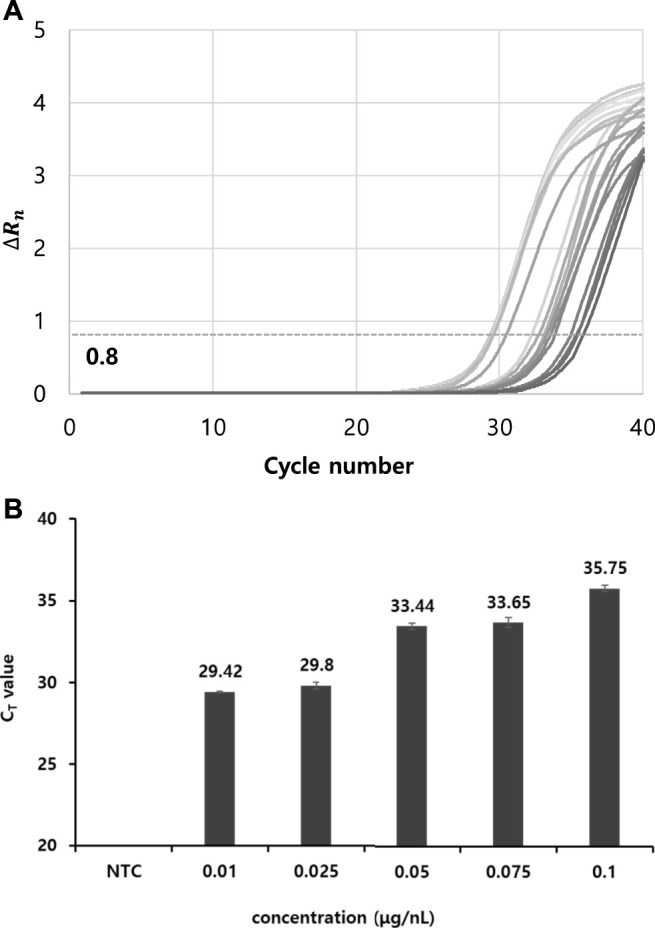
Determination of C_T_ values depending on various TMV template concentration. **(A)** RT-PCR curves with C_T_ numbers. ∆R_n_ threshold was set as 0.8. **(B)** Determination of C_T_ values at template TMV particle concentrations of 0.1 to 0.01 μg/nL. Viral RNA was extracted using a commercial RNA extraction kit, followed by conventional separate RT and PCR reactions. All data were averaged and deviated (bars = S.D., *n* ≥ 3).

TMV was used as a model system to verify thermolysis-based RNA isolation and RT-qPCR. The efficiency of RNA isolation using the commercial kit protocol and thermolysis method were evaluated and compared. Following the extraction of TMV viral RNA using a nucleic acid extraction kit, RT and PCR reactions were carried out according to the manufacturer’s protocol provided by a commercial kit. First, the dependence of the RT-qPCR results on the RNA template concentration was investigated by varying the RNA concentration. As such, the optimal concentration for RT-qPCR was determined (Table 1). The cycle threshold (C_T_), determined when the ∆R_n_ threshold was set at 0.8, tended to increase as the template RNA concentration increased. This is probably because a purified template by RNA extraction kit was used for RT-qPCR reaction whereas various inhibiting chemicals could be generated at a high template concentration when crude viral particle samples are used. In general, the C_T_ ranged from 29 to 35 at RNA concentrations of 0.01–0.1 μg/nL (Figure 1A). An amplification curve was not observed in the negative control without an RNA template and was not infected with the virus, and the lowest RNA concentration that yielded a meaningful C_T_ (29) with RT-qPCR was 0.01 μg/nL (Figure 1B).

**TABLE 1:** C_T_ values at various viral template concentrations. The template was prepared using commercial extraction kit. All data were averaged and deviated (*n* ≥ 3).

Template concentration (μg/nL)	C_T_ value
0	Not detected
0.01	29.42 ± 0.05
0.025	29.80 ± 0.22
0.05	33.44 ± 0.20
0.075	33.65 ± 0.30
0.1	35.75 ± 0.21

### 3.2 Temperature Dependency of Thermolysis and C_T_ Values

**FIGURE 2:**
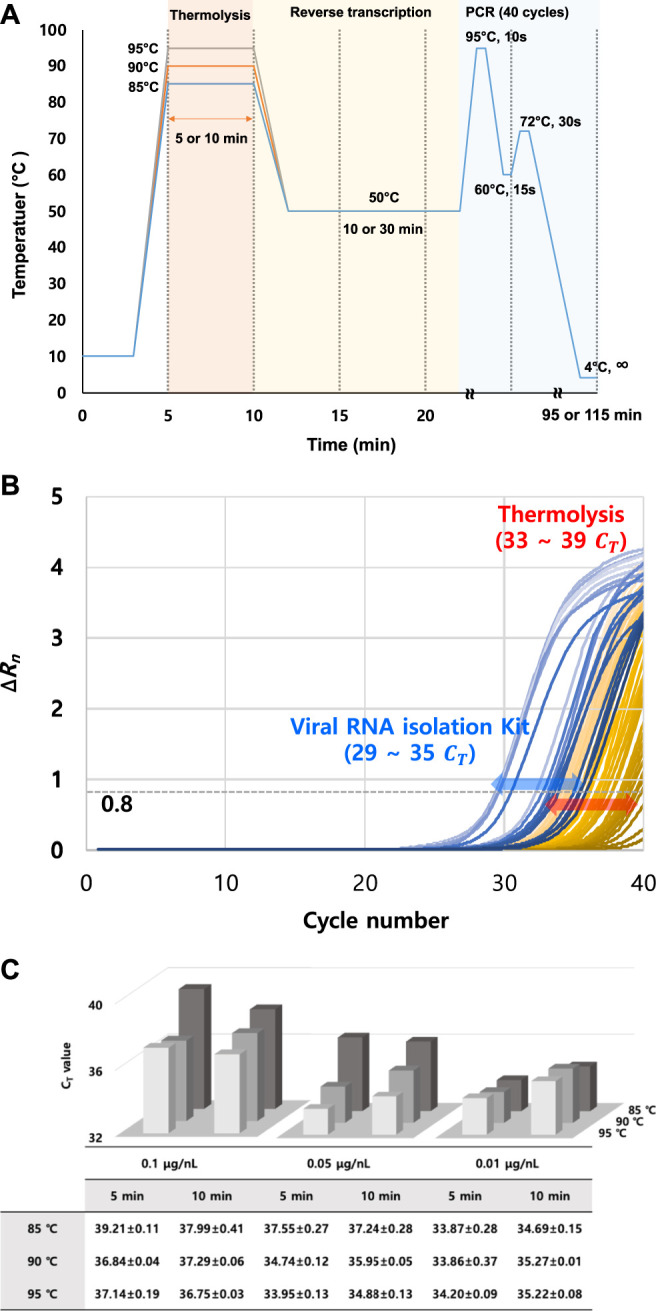
Determination of C_T_ values by varying TMV template concentration and thermolysis conditions. **(A)** Thermolysis-based RT-qPCR protocol optimization by varying thermolysis temperature and time. **(B)** RT-qPCR curve comparison between different RNA isolation methods (RNA isolation kit and thermolysis isolation). C_T_ values were obtained with the ∆R_n_ threshold set as 0.8. **(C)** Summary of obtained C_T_ results under different thermolysis conditions. C_T_ values were obtained by thermolysis-based RNA isolation, followed by conventional separate RT and PCR reactions. All data were averaged and deviated (*n* ≥ 3).

Three different variations in temperature, exposure time, and RNA template concentration were tested for thermolysis before RT-qPCR. This was carried out to verify the performance of the thermolysis method at three different temperatures (at 85, 90, and 95°C), two exposure times (for 5 and 10 min), and three different crude viral particle concentrations in the sample (0.01, 0.05, 0.1 μg/nL). Then, qPCR was evaluated by analyzing C_T_ values in cycles (Figure 2A). Following thermal treatment under 18 different sets of conditions, the crude mixture was directly used as the RT reaction template without a purification step, and the C_T_ was monitored (Supplementary Figures S1–S3). In addition, melting point analysis graphs with no shoulder peak observed were added to the supplementary data (Supplementary Figure S4).

Initially, it was clear that the TMV particles could be detected with RT-qPCR after either thermolysis or commercial RNA isolation. However, slight changes in the C_T_ value depending on thermolysis conditions were observed (Figure 2B). Overall, lower C_T_ values were observed at higher temperatures. This trend was most apparent when the viral concentration was 0.05 μg/nL. The C_T_ differences between 85 and 95°C were highest, around 2.4 to 3.6, whereas temperature did not greatly affect the C_T_ values, with differences of 0.4 and 2.1 at viral particle concentrations of 0.01 and 0.1 μg/nL, respectively. In particular, the temperature dependency on C_T_ values were the weakest at a viral particle concentration of 0.01 μg/nL (Figure 2C).

### 3.3 Effect of RNA Template Concentration on Thermolysis and C_T_ Values

In general, C_T_ values of 33–35 were observed at a viral concentration of 0.01 μg/nL. However, it was increased up to 37 to 39 at 0.1 μg/nL. Interestingly, the average C_T_ values at different thermolysis temperatures and times were lowest at a viral concentration of 0.01 μg/nL. The C_T_ value increased with viral particle concentration. For example, C_T_ value reached a peak of 39 at 0.1 μg/nL with 5 min thermolysis, and reached 37 with 10 min thermolysis. This might be due to the increase in impurities generated from the host during lysis, including tissues, DNA, and viral debris, because the dried viral sample was directly used for thermolysis. These impurities may have acted as inhibitors in the RT-qPCR reaction, thereby resulting in increased C_T_ values. In addition, concentration and temperature had a complex effect on the C_T_ values, as described previously. For example, when the viral particle concentration was as low as 0.01 μg/nL, a dramatic change by the thermolysis temperature was not observed at all.

### 3.4 Exposure Time Dependency on Thermolysis and C_T_ Values

At a viral particle concentration of 0.01 μg/nL, the lowest C_T_ values were obtained at an exposure time of 5 min for all temperatures. As a result of the instability of isolated RNA, long exposure times and high temperatures are likely to degrade RNA templates, leading to higher C_T_ values. With an increase in the temperature and exposure time, there was also an increased release of viral debris into the reaction. Therefore, a clear general tendency of the C_T_-thermolysis relationship was not observed (see the results at 90 and 95°C). There was no significant difference between the C_T_ values at exposure times of 5 and 10 min. For example, at a viral concentration of 0.05 μg/nL, the C_T_ difference was less than 1.0 at all temperatures. This was observed at all the viral concentrations and temperatures.

### 3.5 Determination of Viral RNA Detection Limit

**FIGURE 3:**
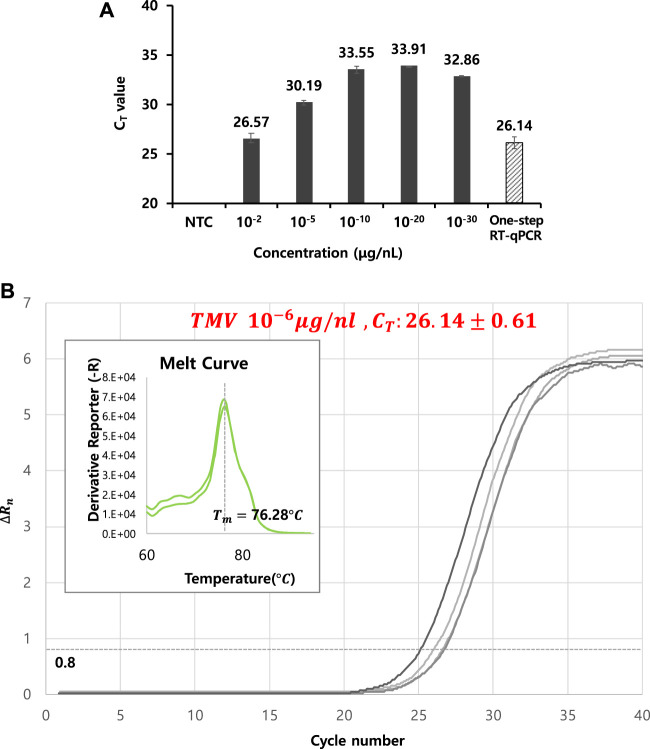
**(A)** Determination of the lowest concentration for viral particle detection using optimized thermolysis conditions at 85°C for 5 min. A specifically designed primer set (see section 2.5 PCR conditions and detection of TMV and CMV) was used for cDNA synthesis, and both RT and PCR reactions were conducted by a one-step process in a single tube. Each C_T_ value was determined against a series of viral particle concentrations of 10^−2^, 10^−5^, 10^−10^, 10^−20^, and 10^−30^ μg/nL (black bar). **(B)** RT-qPCR curves from one-step thermolysis. A viral concentration of 10^−6^ μg/nL was used as a template, and the PCR cycle was programmed by adding two steps: thermolysis for 5 min at 85°C and an RT reaction for 60 min at 50°C prior to the PCR amplification cycles. The obtained C_T_ value was included as slashed bar in Figure 3A for a comparison. All data were averaged and deviated (bars = S.D., *n* ≥ 3).

The lowest concentration for viral particle detection using thermolysis was determined under the optimal thermolysis condition: 85°C for 5 min. A specifically designed primer set described in the experimental section was used for cDNA synthesis, and both RT and PCR reactions were conducted in a single tube by a one-step process. Each C_T_ value was determined against a series of viral particle concentrations of 10^−2^, 10^−5^, 10^−10^, 10^−20^, and 10^−30^ μg/nL, resulting in C_T_ values between 26 and 33 (Table 2). For the 10^−10^ μg/nL sample, the C_T_ value reached 33.55, which was an increase of four cycles compared to the value from the RNA extraction kit-based RT-qPCR. A similar range of C_T_ values was maintained up to the 10^−30^ μg/nL diluted sample. These results suggested that the limit of detection under thermolysis conditions was approximately 10^−5^ and 10^−10^ (Figure 3A).

**TABLE 2:** Determination of limit of detection concentration by varying the tobacco mosaic virus template concentration. All data were averaged and deviated (*n* ≥ 3).

Template concentration (μg/nL)	C_T_ value
0	Not detected
10^–2^	26.57 ± 0.47
10^–5^	30.19 ± 0.24
10^–10^	33.55 ± 0.35
10^–20^	33.91 ± 0.02
10^–30^	32.86 ± 0.07

### 3.6 Feasibility of One-Step Thermolysis RT-qPCR

Next, the feasibility of combining thermolysis and RT-qPCR in a single tube using a programmed PCR cycle was investigated against TMV. A mixture solution containing viral particles, constituents of the RT reaction, constituents of the qPCR reaction, and specific primers was prepared and reacted in a PCR thermocycler. The PCR cycle was programmed by adding two steps of thermolysis for 5 min at 85°C and an RT reaction for 60 min at 50°C prior to the PCR amplification cycles. When a 10^−6^ μg/nL viral particle concentration was used as the template, cDNA was successfully synthesized by RT and amplified by qPCR, with a C_T_ value of 26.14 ± 0.61 (Figure 3B). Also, the amplified DNA products were again separated by gel electrophoresis analysis and verified to confirm the correct amplification.

### 3.7 Optimization of One-Step Thermolysis RT-qPCR

**FIGURE 4:**
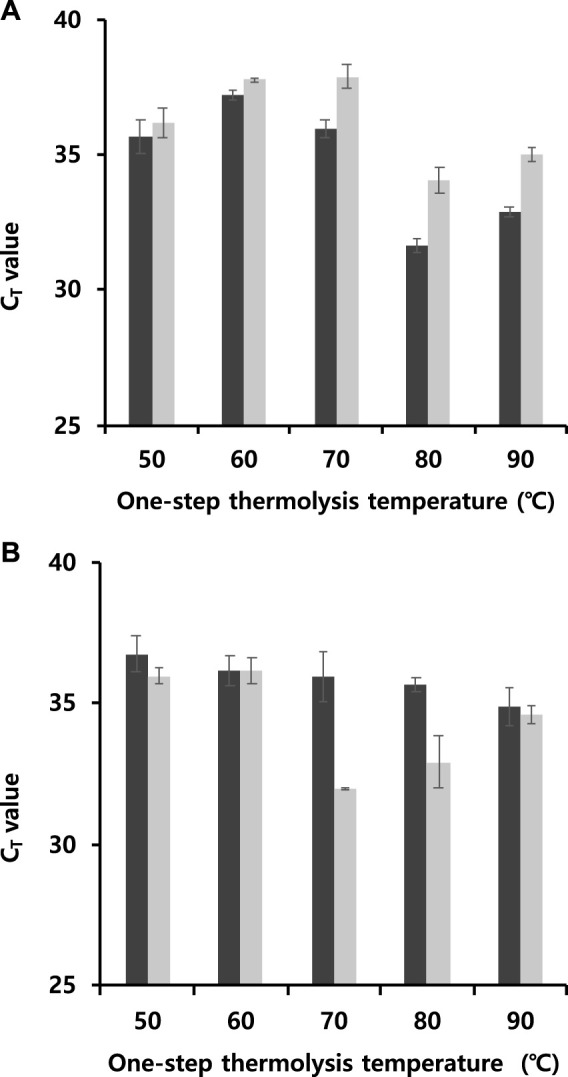
Determination of C_T_ values to reduce total operational time by reducing the difference between the thermolysis and RT temperatures at a viral particle concentration of 10^−6^ μg/nL. **(A)** TMV and **(B)** CMV (Black bar: 5 min, grey bar: 10 min). The thermolysis temperature varied from 50 to 90°C for five or 10 min, followed by RT reaction at same temperature. All data were averaged and deviated (bars = S.D., *n* ≥ 3).

The operational process for one-step thermolysis RT-qPCR was optimized by reducing the total operation time. In this case, CMV was tested to apply the one-step protocol to other viral particles. The most time-consuming step in the developed protocol was the RT reaction, which was set to 80 min. The initial step of 5 min exposure time for thermolysis at 85°C included additional time, required to lower the temperature to the optimum RT temperature of 50°C, and needed to maintain this temperature for 30 min or more. The first attempt to reduce the total operation time was carried out by reducing the difference between the thermolysis and RT temperatures with the viral particle concentration fixed at 10^−6^ μg/nL. The thermolysis temperature was optimized by varying the temperature from 50 to 90°C for five or 10 min, followed by RT reaction at same temperature. One-step thermolysis in programmed PCR against TMV was most effective at 80°C for 5 min, while the optimal condition against CMV was found to be 70°C for 10 min (Figures 4A,B). However, no significant difference of three cycles or more was observed at any thermal treatment time for either virus, indicating feasibility of the thermolysis protocol at lower temperatures.

Then, the feasibility of synchronizing thermolysis and RT at 60°C was verified by programming a continuous isothermal reaction in a thermocycler, thereby reducing the total exposure time up to 5 and 10 min. Interestingly, when the total time of thermolysis and RT reaction was 10 min against either TMV or CMV particles, the C_T_ was less than 33 (Table 3). This suggested that not only was thermolysis successful at 60°C, but also that the RT reaction was effective even under 10 min, although a slight C_T_ increase of 4–five cycles was observed compared to values at 85°C thermolysis (Figure 3B). Moreover, TMV was detected with C_T_ value of 30.49 ± 0.06 at a total reaction time of 5 min. These findings confirmed that continuous one-step thermolysis, RT, and PCR could be performed in a single PCR tube using a single thermocycler.

**TABLE 3:** Results of one-step detection of tobacco mosaic virus (TMV) and cucumber mosaic virus (CMV) in one tube. All data were averaged and deviated (*n* ≥ 3).

Viral targets	Thermolysis + reverse transcription time^a^	C_T_ values
TMV	5 min	30.49 ± 0.06
	10 min	32.68 ± 0.11
CMV	5 min	not determined
	10 min	32.42 ± 0.32

a Temperature for thermolysis and reverse transcription reaction: 60°C.

### 3.8 dPCR-Based Viral Particle Detection

**FIGURE 5:**
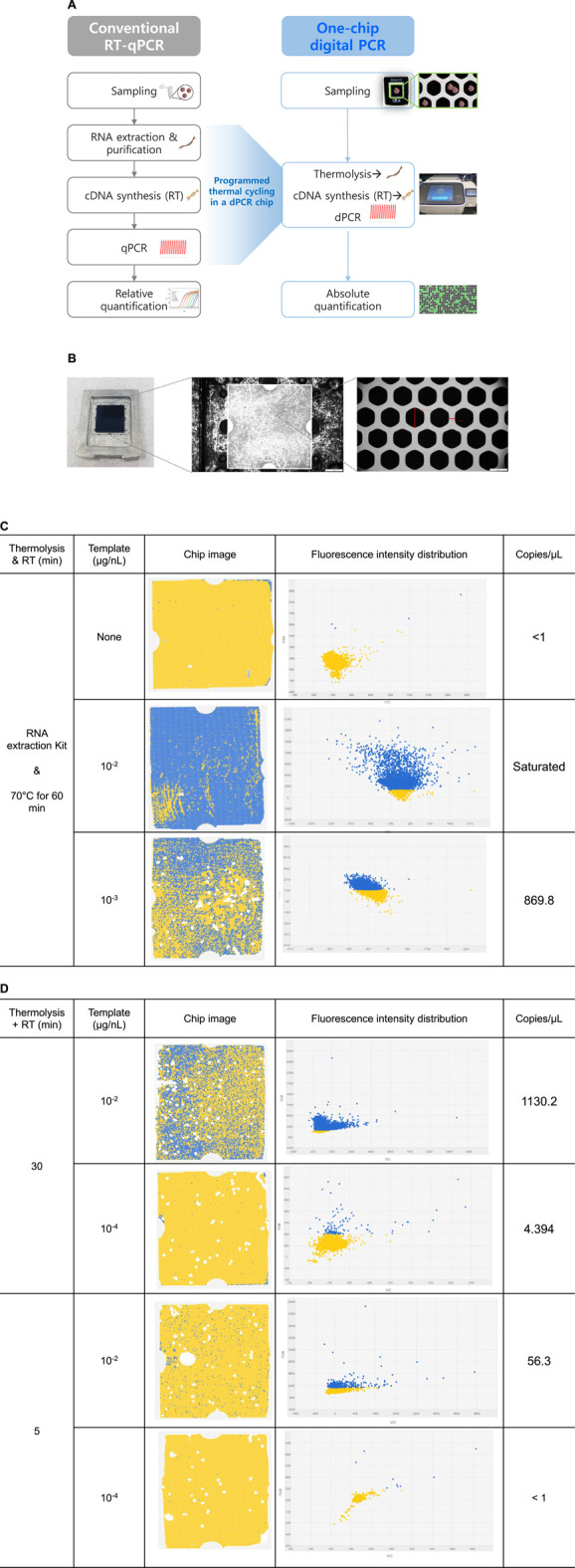
Digital PCR analytical protocols and CMV detection results with chip images and fluorescence intensity distribution. **(A)** Overall digital PCR analysis scheme compared to conventional RT-qPCR analysis. **(B)** Photograph of the QuantStudio 3D digital PCR chip (10 × 10 × 0.3 mm^3^) with 20,000 nanoscale through-hole PCR wells, and optical microscope image of through-hole PCR wells. The diagonal length of through-hole is about 60 μm and the width of sidewall is about 18 μm. **(C)** Digital PCR chip analysis images. CMV concentrations of 0, 10^−2^, and 10^−3^ μg/nL. Fluorescence intensity distribution at each CMV template concentration. For RNA isolation, a commercial RNA extraction kit was used, and RT reactions were conducted at 70°C for 60 min. **(D)** One-chip digital PCR results of CMV detection with dPCR chip analysis images. Simultaneous thermolysis and RT reactions were conducted at 70°C for either 30 min or 5 min, and CMV concentration were set at either 10^−2^ μg/nL or 10^−4^ μg/nL.

Viral particle analysis was performed using dPCR to verify whether the one-step analysis protocol obtained from RT-qPCR analysis was appropriate for dPCR assay. A ThermoFisher QuantStudio 3D digital PCR system was used, in which the DNA concentration of a sample could be quantified with a dPCR chip containing 20,000 chambers of less than 1 nL (Figure 5A). The application of a thermolysis-based protocol in sub-nanoliter-sized partitioned chambers has a great advantage in that partitioning has both concentration and purification effects, minimizing inhibition by lysis debris and RT reaction components. Considering the size of a single dPCR chamber (approximately 60 μm in diameter and 300 μm in height, Figure 5B), the sphere-shaped CMV particles with 20 nm diameter would be more appropriate for positioning in a dPCR chamber than the rod-shaped TMV particles with 300 nm length and 10 nm width. Therefore, CMV particles were used as model viral particles for the dPCR analysis.

The cDNA synthesized by RT reaction (70°C, 60 min) from CMV particles using an RNA extraction kit was used as a qPCR template for comparison, and the results showed a C_T_ value of 35.47 ± 0.33. This corresponded to the observed negative control values. In the dPCR system, a negative control without viral particles resulted in an output of less than 1 copy/μL. Analysis with viral concentrations of 10^−2^ and 10^−3^ μg/nL resulted in a saturated and 869.8/μL concentration output, respectively (Figure 5C). The concentration of RNA copies from the 10^−2^ μg/nL sample was interpreted as saturated because the number of positive chambers was too large to be quantified using Poisson statistics. It was predicted that the viral particle concentration in 10^−3^ μg/nL yielded approximately 8.7×10^2^ copies RNA/μg viral particles based on the amplification output value of 869.8 copies/μL in the dPCR analysis.

### 3.9 One-Chip Digital PCR Based on Thermolysis Protocol

It was confirmed that the detection of viral was feasible with meaningful output through dPCR. Based on previous results, the one-step RT-qPCR protocol was applied to dPCR analysis to verify the one-chip digital PCR protocol. Although the optimal conditions of 85°C thermolysis for 5 min were secured as one-step assay conditions in RT-qPCR assay, thermolysis in the one-chip dPCR system was performed at 70°C to maintain activation of the reverse transcriptase, which was more critical in one-chip dPCR assay. Therefore, a continuous thermolysis-RT reaction cycle for either 30 or 5 min was introduced prior to the PCR cycle, and the concentration output results were compared at 10^−2^ μg/nL and 10^−4^ μg/nL viral particle templates, respectively.

At a viral particle concentration of 10^−2^ μg/nL, output values of 1,130.2 and 56.3 copies/μL were calculated for 30 and 5 min thermolysis-RT reactions, respectively (Figure 5D). This suggested that longer thermolysis-RT reaction time led to higher efficiency of RNA separation and cDNA conversion from the viral particles, resulting in a higher output. Meanwhile, output values of 4.4 and less than 1 copies/μL concentration were obtained at 30 and 5 min thermolysis-RT reactions at 10^−4^ μg/nL concentration, respectively. This suggested that the limit of detection for the 5 min reaction time was 10^−4^ μg/nL. However, no cDNA amplification was obtained in any of these four reaction conditions when the same one-chip dPCR conditions were applied to RT-qPCR analysis, suggesting that it would be impossible to detect the viral particles with qPCR under conditions that were suitable for one-chip dPCR conditions. In conclusion, the results that 10^2^ to 10^3^ copies RNA/μg ranges of viral particles, which were calculated by multiplying the viral particle concentration with the output copies/μL, could be detectable even in a 5 min reaction, which supports the hypothesis that the one-chip dPCR protocol can efficiently detect viral particles quantitatively.

## 4 Conclusion

This study developed a thermolysis-based RT-qPCR protocol to overcome the laborious and time-consuming process of conventional RT-PCR reactions by circumventing RNA isolation and purification steps. TMV and CMV were tested as model viruses using this protocol, and were directly detected. Of all thermolysis conditions, the lowest C_T_ value was obtained at 85°C thermolysis for 5 min with 0.01 μg/nL of viral particles. Compared to the C_T_ values obtained using a commercial RNA extraction kit, the TMV concentrations of 0.1, 0.05, 0.1 μg/nL produced C_T_ differences of 1.0–3.5, 0.5–4.1, and 4.4–5.9, respectively. Under all thermolysis conditions, the C_T_ values were approximately 33–39, which was about four cycles higher than those resulting from a commercial RNA extraction kit (Figure 2B). It was demonstrated that viral particles could be detected with distinct thresholds through RT-qPCR using the thermolysis method.

**FIGURE 6:**
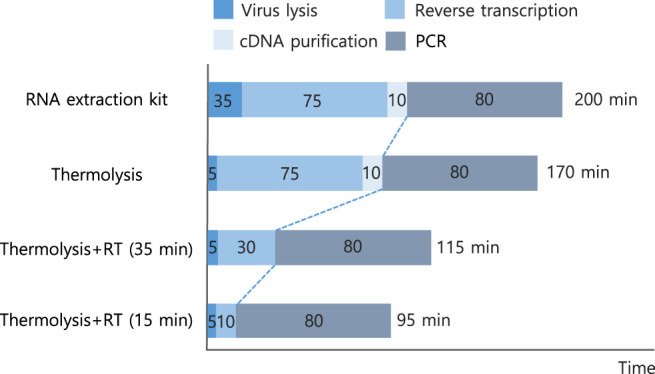
Summary of the developed thermolysis-based one-step qPCR protocol and comparison of operation time. Numbers in each bar indicate each operational time in min. Details of operational time and C_T_ values are listed in Table 4.

The protocol focused on a simple and rapid virus detection method. By optimizing thermolysis conditions, such as temperature, exposure time, and template concentrations, an effective RT-qPCR protocol was developed, achieving a significant decrease in the total operational time compared to conventional methods (Figure 6). The continuous one-step thermolysis of viral particles and an RT reaction at 60°C for 5 min followed by PCR reactions within a single tube were successful in detecting viral particles within 95 min of total operational time. A total of 105 min in operational time could be saved compared to that of commercial kit-based RNA isolation and quantitative RT-qPCR methods (Table 4). Additionally, there was considerable improvement in the C_T_ value, which was 20.72 ± 0.69, although the threshold was set to a value of 0.4, as the one-step RT-qPCR resulted in a relatively low amplification intensity compared to those of previously optimized protocols.

**TABLE 4:** Summary of the developed thermolysis-based qPCR protocol and comparison of operation time and C_T_ values. All data were averaged and deviated (*n* ≥ 3).

Protocol	RNA extraction	Reverse transcription	cDNA purification	PCR	Total time (min)	C_T_ value (cycles)
RNA extraction kit	35 (Extraction kit)	75	10	80	200	29.42 ± 0.05
Thermolysis	5 (thermolysis)	75	10	80	170	33.87 ± 0.29
Thermolysis + RT-PCR (35 min)	5 (thermolysis)	30	—	80	115	21.95 ± 0.44^a^
Thermolysis + RT-PCR (15 min)	5 (thermolysis)	10	—	80	95	20.72 ± 0.69^a^

a C_T_, values were determined at thresholds of 0.4 due to lowered intensity.

The developed method presents opportunities to be further refined, decreasing the total time to less than 1 h. The only protocol that was not optimized or engineered in this study was that of PCR cycling, which was set as a 10-10-35 s process with 40 cycles. cDNA amplification through PCR therefore occupied most of the viral RNA detection time, considering the shorted RNA extraction and cDNA synthesis step. There is ample scope for shortening the viral RNA detection time by shortening the PCR cycle. For this purpose, future optimization studies are needed. For example, the design of an optimal oligonucleotide primer set for amplification and the selection of the ideal amplification region and length could shorten the overall process. In addition, optimization of PCR cycle time and reaction conditions suitable for one-step and dPCR systems is required.

The developed protocol was applied to one-step dPCR, which allowed the detection of 1,130.2 RNA copies/µL using 10^−2^ μg/nL of viral particles with a 30 min thermolysis-RT reaction at 70°C (Table 5). The protocol developed by optimizing RT-qPCR analysis was verified and applied to dPCR analysis. Not only did the dPCR technique provide a much higher precision and accuracy in quantifying nucleic acids than that of the qPCR, but it also allowed for suitable reaction conditions in a one-chip assay. Considering the physical shape of the dPCR chip, one of the benefits of this system is the increase in concentration and purification effect by partitioning (Basu, 2017). In addition, silicon-based dPCR has a much higher heat transfer and thermal controllability in sub-nanoliter-sized chambers, resulting in faster thermal changes during PCR cycles. Because the PCR reaction depends on thermal changes during the amplification cycle, most of the operational time relies on thermal ramping steps rather than each reaction, so efficient thermal changes speed up the process. For the conventional PCR reaction, components such as dNTPs, polymerase, oligomers, and other reagents were mixed in a 10–20 μL reaction volume. However, this could be dispersed into less than 1 nL of reaction volume under dPCR conditions, providing much less possibility of interference by reaction elements. This effective concentration and purification by partitioning was optimal in continuous RT and PCR reactions in a single dPCR chamber because fewer interfering and inhibiting elements in different reaction systems could be effectively partitioned in a single dPCR chamber, resulting in higher RNA and cDNA yields and amplification.

**TABLE 5:** One-chip analysis results for cucumber mosaic virus particle detection. Chip image and fluorescence intensity distribution are shown in Figure 6.

Protocol	Thermolysis (min)	Reverse transcription (min)	Template (μg/nL)	Concentration (copies/μL)
RNA extraction kit	35	60	0	<1.0
	35	60	10^–2^	Saturated
	35	60	10^–3^	869.8
One-chip assay	Thermolysis	30	10^–2^	1,130.2
		5	10^–2^	56.3
		30	10^–4^	4.4
		5	10^–4^	<1.0

Also, the precision in dPCR was well known to be limited by the Poisson statistics of which the total number of template molecules in the analyzed sample. This means that the lower prevision could be obtained as the volume of the viral particle increases in a limited reaction volume. When applied to RT-qPCR, the size of the virus is not a big problem because there are few spatial restrictions, but when applied to digital PCR, the size of the virus is also one of the important variables. However, the chip volume can be controllable depending on the size of analyzing viral particles. The sensitivity of virus detection due to the volume effect may be affected, but since the size of the digital PCR chamber is approximately 60 μm, it is possible to sufficiently detect viruses corresponding to 20–100 nm size. This was confirmed through digital PCR analysis results for TMV corresponding to 18 nm × 300 nm, which showed analysis results at a level similar to that of CMV at 20 nm. To efficiently detect viral RNA, it is important to develop analysis protocols simultaneously. This indicates the necessity of studying reverse transcriptase and DNA polymerase enzymes, which are critical factors in cDNA synthesis and amplification. In addition, most commercial enzymes and supplementary element concentrations are not suitable for the chamber size of the dPCR system, and will require optimization for efficient viral RNA detection and diagnosis. Currently, RT-qPCR probes, such as Progema company’s GoTaq^®^ Probe qPCR and RT-qPCR system or myPOLS company’s Volcano3G^®^ RT-PCR master mix, operate in a single step. In addition, commercial kits that can accurately detect viral particles with a high speed as the goal of this study are emerging due to the development of hot-start polymerase enzymes that optimize the different reaction conditions of RT and PCR such as temperature, buffer, and pH.

Various techniques have been developed for detecting viral RNA. For these technological developments, analyzing equipment, detection protocols and data analysis should proceed together. The precision and accuracy of viral RNA diagnosis are important along with the speed of the method. In particular, the qPCR technologies verified in this study can be utilized as a technology that enables mankind to cope with and overcome pandemic situations by pathogens in the future.

## Data Availability

The original contributions presented in the study are included in the article/Supplementary Material, further inquiries can be directed to the corresponding authors.
