# Supplementary material for: One-Step RT-qPCR for Viral RNA Detection Using Digital Analysis
Source: Front Bioeng Biotechnol. 2022 Mar 7;10:837838. doi: 10.3389/fbioe.2022.837838 (PMC8948435; doi:10.3389/fbioe.2022.837838)
Supplement: Supplementary file 1 [file DataSheet1.docx]

Supplementary Material


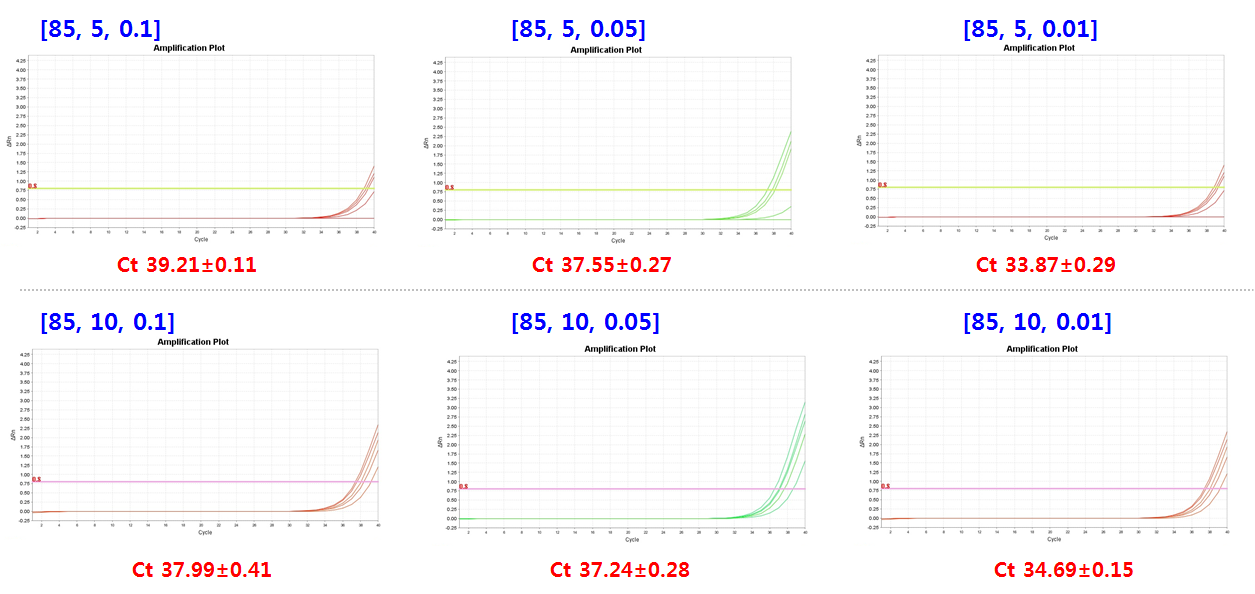


**Figure S1.** qRT-PCR curves at 85 °C [temperature, time, concentration]


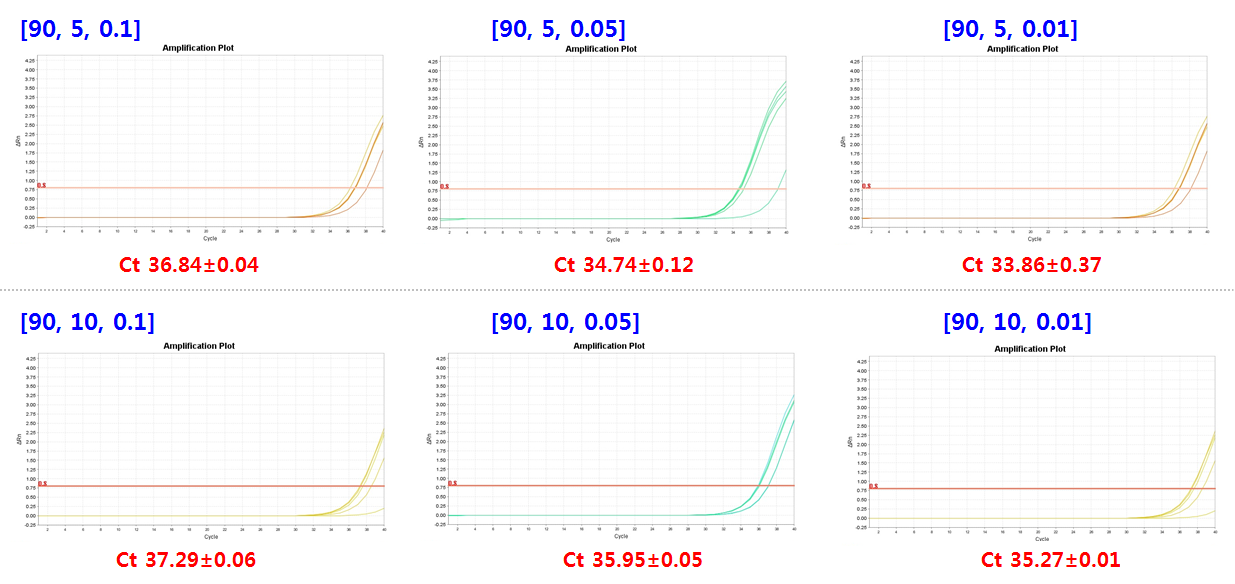


**Figure S2.** qRT-PCR curves at 90 °C [temperature, time, concentration]


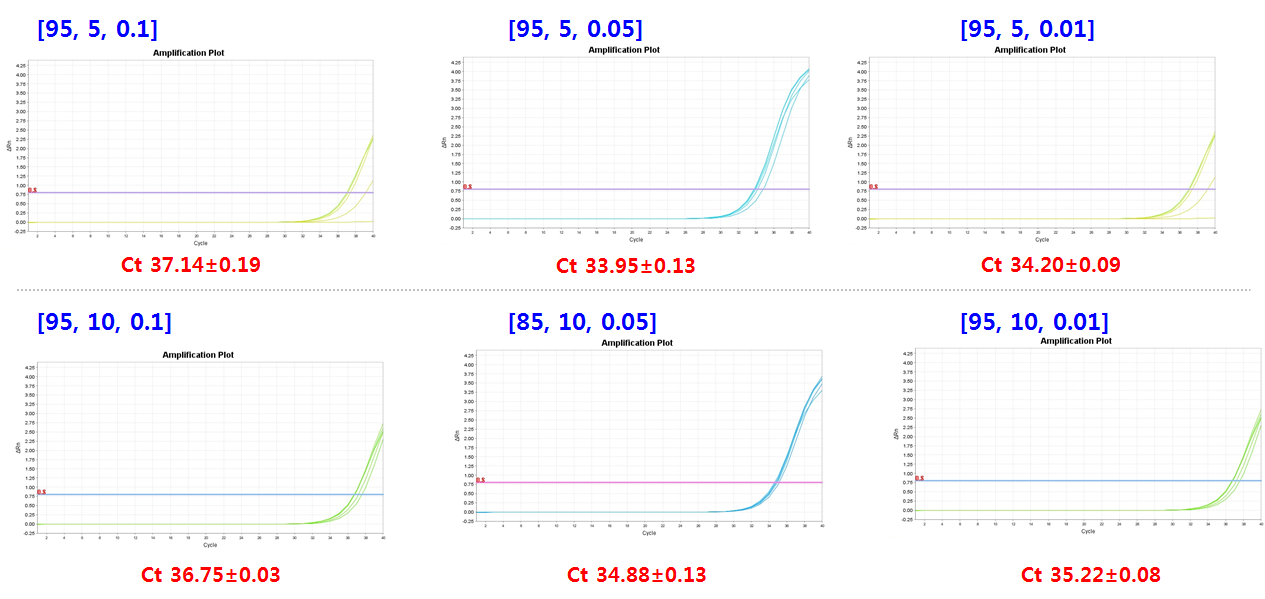


**Figure S3.** qRT-PCR curves at 95 °C [temperature, time, concentration]


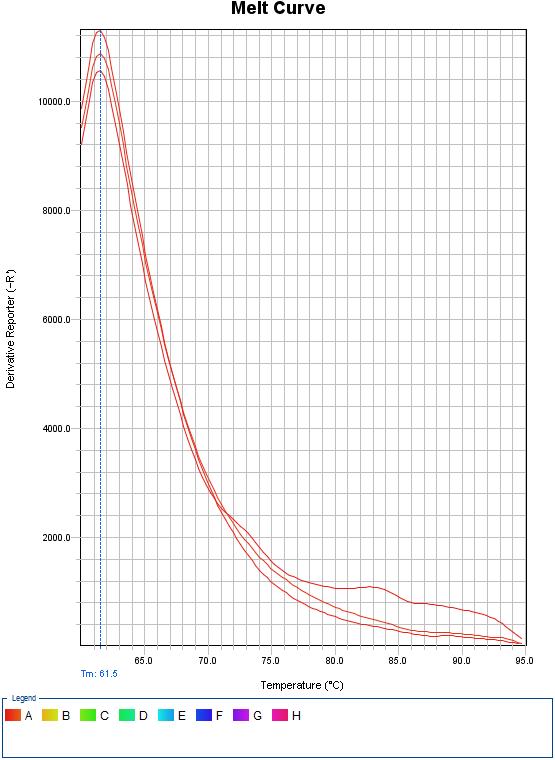

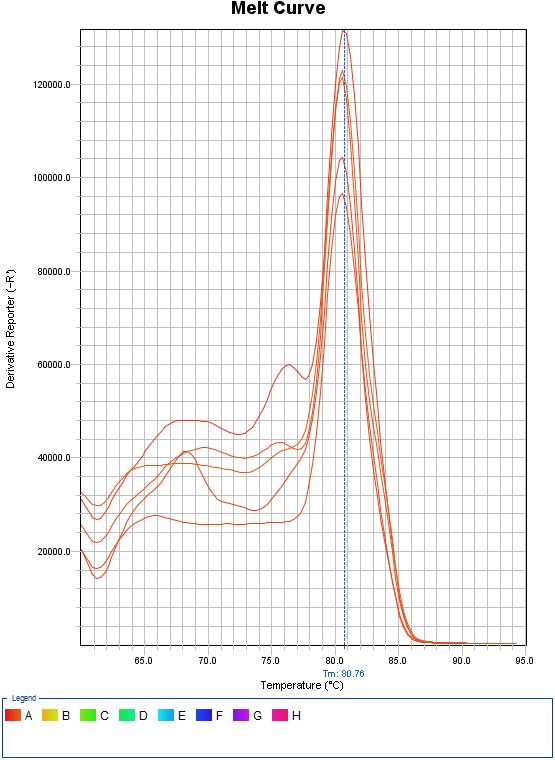

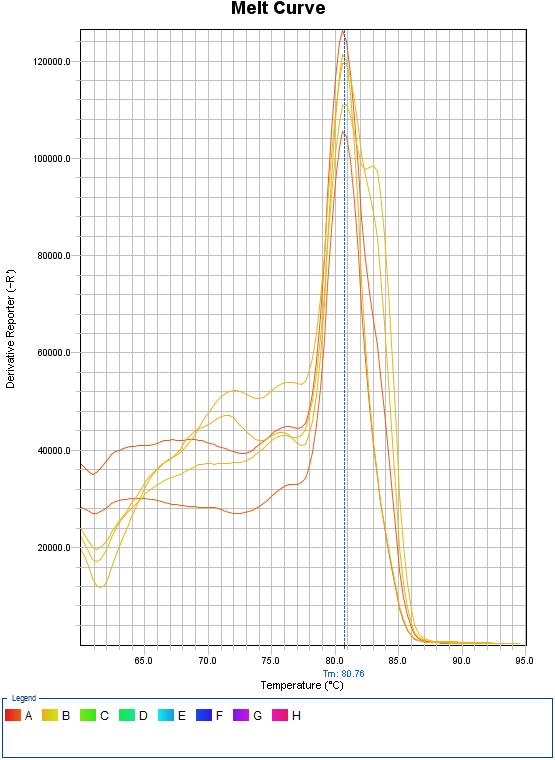

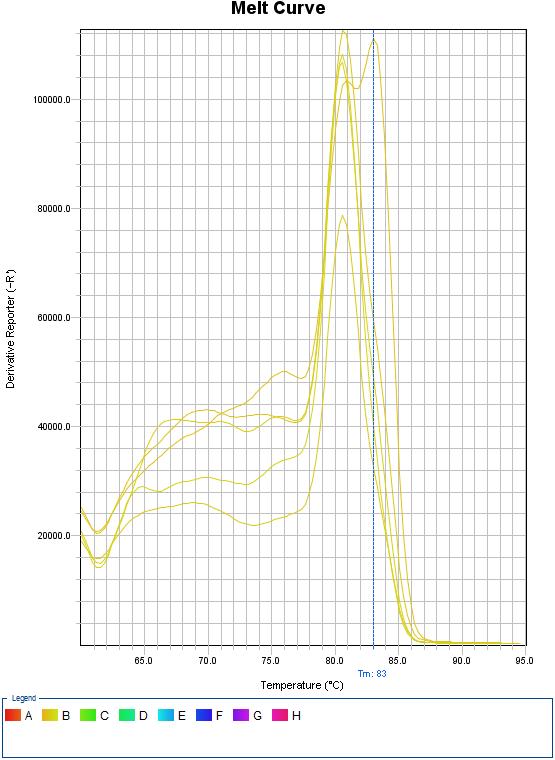

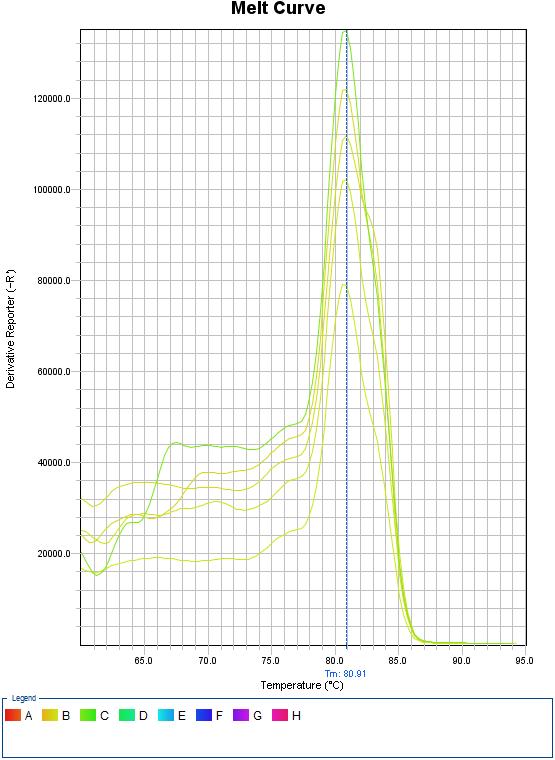

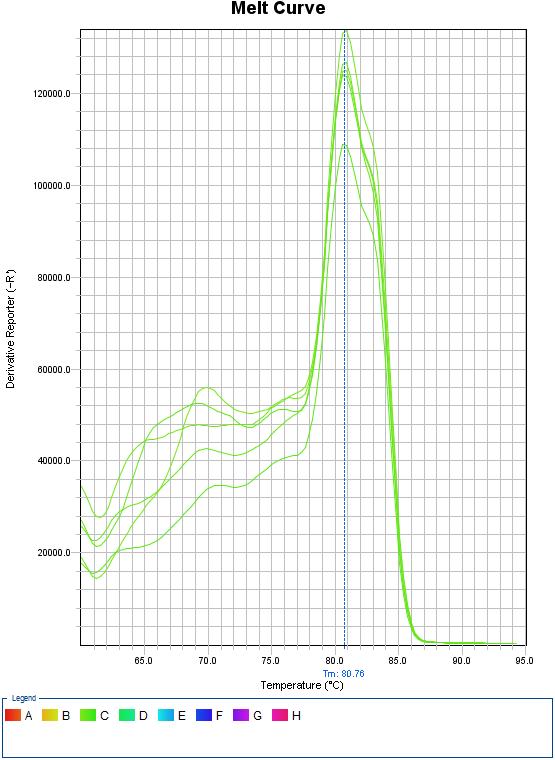


**Figure S4.** Melting curve analysis for RT-qPCR optimization. Melting curves at quantitative PCR against NCT and 0.1 μg/nL. 0.075 μg/nL, 0.05 μg/nL, 0.025 μg/nL, and 0.01 μg/nL of CMV template concentration.
